# *QuickStats:* Percentage* of Children and Adolescents Aged ≤17 Years Who Have Experienced a Specified Stressful Life Event,^†^ by Type of Event and Family Income^§^ — National Health Interview Survey,^¶^ United States, 2021

**DOI:** 10.15585/mmwr.mm7229a7

**Published:** 2023-07-21

**Figure:**
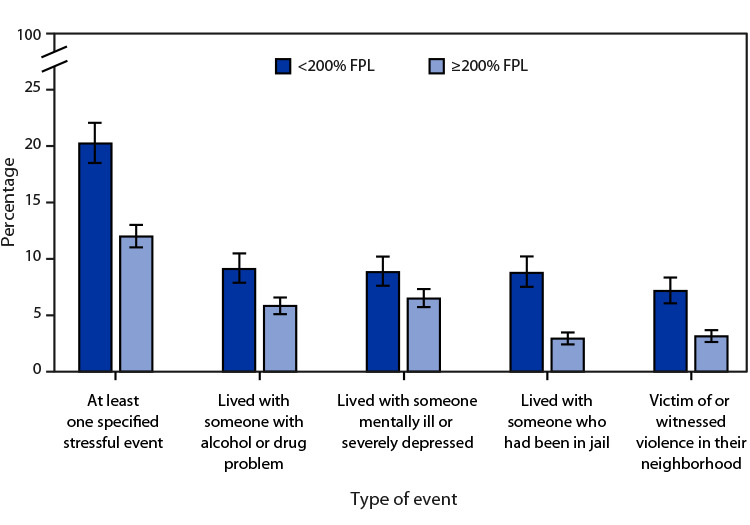
In 2021, 20.2% of children and adolescents in families with incomes <200% of FPL and 12.0% of those in families with incomes ≥200% of FPL had experienced at least one specified stressful life event. Children and adolescents in families with incomes <200% of FPL were more likely than those in families with incomes ≥200% of FPL to have had the following experiences: lived with someone with alcohol or drug problems (9.1% versus 5.8%); lived with someone who was mentally ill or severely depressed (8.8% versus 6.5%); lived with someone who had been in jail (8.8% versus 2.9%); or been the victim of or witnessed violence in their neighborhood (7.2% versus 3.1%).

**Source:** National Center for Health Statistics, National Health Interview Survey, 2021. https://www.cdc.gov/nchs/nhis/index.htm

For more information on this topic, CDC recommends the following link: https://www.cdc.gov/violenceprevention/aces/index.html

